# Glucocorticoid response to both predictable and unpredictable challenges detected as corticosterone metabolites in collared flycatcher droppings

**DOI:** 10.1371/journal.pone.0209289

**Published:** 2018-12-20

**Authors:** Kevin Fletcher, Ye Xiong, Erika Fletcher, Lars Gustafsson

**Affiliations:** 1 Department of Ecology and Genetics, Animal Ecology, Uppsala University, Uppsala, Sweden; 2 Biomedicine Centre, Pharmacuetical Biosciences, Uppsala University, Uppsala, Sweden; University of Pretoria, SOUTH AFRICA

## Abstract

In most vertebrate animals, glucocorticoid hormones are the chief mediators of homeostasis in response to ecological conditions and as they progress through their lifecycle. In addition, glucocorticoids are a major part of the stress response and stress induced elevations of the hormone can make it difficult to assess glucocorticoid secretion in response to changes in life-stage and current environmental conditions in wild animals. Particularly when quantifying circulating levels of glucocorticoids in the blood which fluctuate rapidly in response to stress. An alternative method of quantifying glucocorticoids is as hormone metabolites in faeces or urine giving a historical sample related to the gut passage time and urinary tract that is less sensitive to stressful events which cause spikes in the circulating hormone level. Although the concentration of glucocorticoid metabolites are influenced by faecal mass thereby potentially affecting any differences in hormone metabolites detected amongst samples. In the present study, we aimed to detect changes in levels of corticosterone, the primary bird glucocorticoid, in relation to the phase of reproduction, in a breeding population of collared flycatchers by sampling corticosterone metabolites in droppings. We also tested how corticosterone metabolite concentrations were affected by ambient temperature and related to body condition in adult birds. Our results indicate that the upregulation of corticosterone between incubation and nestling feeding in female birds is crucial for successful reproduction in this species. Also, females appear to downregulate corticosterone during incubation in response to lower ambient temperature and poorer body condition. Our results did not indicate a relationship between dropping mass and corticosterone metabolite concentrations, which suggests that our findings were linked to the regulation of corticosterone in response to predictable and unpredictable challenges.

## Introduction

In most vertebrates, exposure to changes in environmental conditions or stressful situations results in the stimulation of the hypothalamus-pituitary-adrenal (HPA) axis. Initiation of the HPA-axis results in higher levels of glucocorticoids (GCs) in the blood, which then mediate many alternative metabolic pathways and behaviours. The metabolic pathways initiated by elevated GCs mobilize energy stores by the catabolism of proteins [1] and also regulate glucose levels [2] thereby making energy available to facilitate behaviours in the short-term. However, if elevated levels of GCs are prolonged, negative effects can include abandonment of reproduction [3], severe debilitation, or even death[3]. In addition to short-term elevated GC responses to unpredictable stimuli, GCs are released at higher operating levels during periods that are predictably energy demanding, for example, reproduction [4–7]. Moreover, GCs are the principal mediators of allostasis [7] which describes all of the physiological mechanisms that are involved in maintaining homeostasis [8] as an animal proceeds through different life-history stages and responds to the environment [9].

In altricial birds, there are three distinct phases of reproduction: egg laying, incubation and feeding nestlings until independence. Each stage has different energetic requirements [10] as well as the potential for inflated energy costs in response to adverse environmental conditions [11]. For instance, the peak of energy demand during the breeding period is most often considered to be during the nestling feeding stage [11], while the level of energy expended during incubation is thought to be negligible [12]. However, below thermos-neutrality (typical of temperate regions) the energy demand of incubation exceeds that of a non-incubating bird at rest which is tied to current environmental conditions [13,14]. A popular method used to detect how wild animals respond to changes in life stage [15–18] and unpredictable challenges such as inclement weather [19] is to quantify the glucocorticoid response. For example, in bird studies it has been shown that corticosterone—the primary bird glucocorticoid—is upregulated during the breeding season to meet energy requirements which has a positive effect on reproductive success [16,17,20–22]. However, in response to adverse weather conditions and reduced food availability elevated corticosterone levels can also signify stress [11,23,24] and thus is a potential proxy of current body condition. In light of these competing glucocorticoid functions, care should be taken to consider both the lifecycle and current environmental conditions being experienced by the animal when assessing corticosterone secretion.

Given that corticosterone is produced almost immediately after stimulation resulting in increased hormone in the blood, the most common technique to quantify corticosterone level is in blood plasma, which provides a measurement from a single time point [25]. Other methods, such as quantifying corticosterone metabolites in faeces or urine samples has the benefit of being less susceptible to handling stress and provides a historical measurement linked to the period of gut passage and urinary tract [26,27]. It has been shown in studies using captive birds that an induced stress response stimulated by adrenocorticotropic hormone was detectable as corticosterone metabolites in droppings between 1 and 2 hours after stimulation [9,28–30]. Moreover, by reducing the effect of stress-induced spikes on circulating levels of corticosterone, corticosterone metabolites may in-fact provide a better estimate of the current level of corticosterone in response to daily activity, experimental treatments and long-term corticosterone profiles [27]. However, quantifying corticosterone metabolites in faeces can be problematic because the concentration detected is linked to faecal mass which is influenced by food consumption that changes due to environmental factors and current life stage [30].

In this study, we aimed to detect changes in corticosterone metabolite concentrations in the droppings of collared flycatchers during the breeding season. First, we tested the relationship between dropping mass and corticosterone metabolite concentration. If dropping mass influenced the concentration of corticosterone metabolite detected, we would have expected a negative correlation. Next, we compared the level of corticosterone metabolites in female droppings during incubation and nestling feeding to determine if the anticipated increase of corticosterone in response to increased energy demand was detectable. We also tested the relationship between corticosterone metabolite concentrations and current ambient temperature as well as body condition in adult birds.

## Materials and methods

### Study site

We collected data in 2015 and 2016 from a nest-box population of collared flycatchers in the southern part of the island of Gotland in the Baltic Sea (57^0^10^’^ N, 18^0^20^’^ E). The nest boxes, some of which have been monitored since 1980 [31], are set up in mixed deciduous woodland, the preferred habitat of the collared flycatcher [32]. The collared flycatcher readily breeds in nest boxes often returning to the same nesting site in subsequent years and it is a species that breeds undeterred by human presence. The birds start to arrive from the wintering grounds in sub-Saharan Africa towards the end of April when courtship begins, which involves a male attempting to attract a female to a preferred nesting site [33]. Once a breeding pair establishes, the egg-laying phase usually occurs in the second week of May. The females lay between three and nine eggs that she solely incubates for approximately 12 days [32]. To determine hatch date, we visited the nest daily 11 days after the onset of incubation. There is some evidence to suggest that the male birds supplement the female with food during incubation and both of the parents provide care for the young.

### Data collection

We caught adult birds in the nest-box by partially covering the entry hole with a clear piece of soft plastic. The plastic was fixed on the inside of the box with two pins allowing the bird to enter the box unhindered but prevented the bird from leaving. This method of trapping had the added benefit of trapping both parents during the same trapping event. Traps were checked and removed after 15 minutes. Female birds were sampled twice, both during incubation and during the nestling feeding stage, while males were only tested once during the nestling feeding stage. We sampled 45 females both during incubation and nestling feeding, 24 in 2015 and 21 in 2016. While we sampled 61 male and female pairs during nestling feeding, 25 in 2015 and 36 in 2016. We quantified corticosterone as metabolised corticosterone in the droppings of collared flycatchers. The droppings were collected during data collection of the female bird five days after the start of incubation, and then both the male and the female simultaneously five days into the nestling feeding stage. We collect a range of morphometric data from the birds in close proximity to their nest-box which takes about 10 minutes. Dropping samples were collected from birds that produced a dropping within 10 minutes after removal from the nest-box. Following excretion, the bird droppings were immediately transferred to a 1.5 ml eppindorf tube then homogenised (mixed with a spatula) and kept on ice before being stored in a -80°C freezer. We stored the samples in a -80°C freezer following collection then transferred them on dry-ice to a -80°C freezer at the final storage destination before analysis. The data upon which this study is based, have been obtained following the Swedish guidelines for work on natural populations, and under licenses and permits from the Swedish Ringing Centre and Swedish National Board for Laboratory Animals, Stockholm.

### Hormone analysis

**Fig 1:**
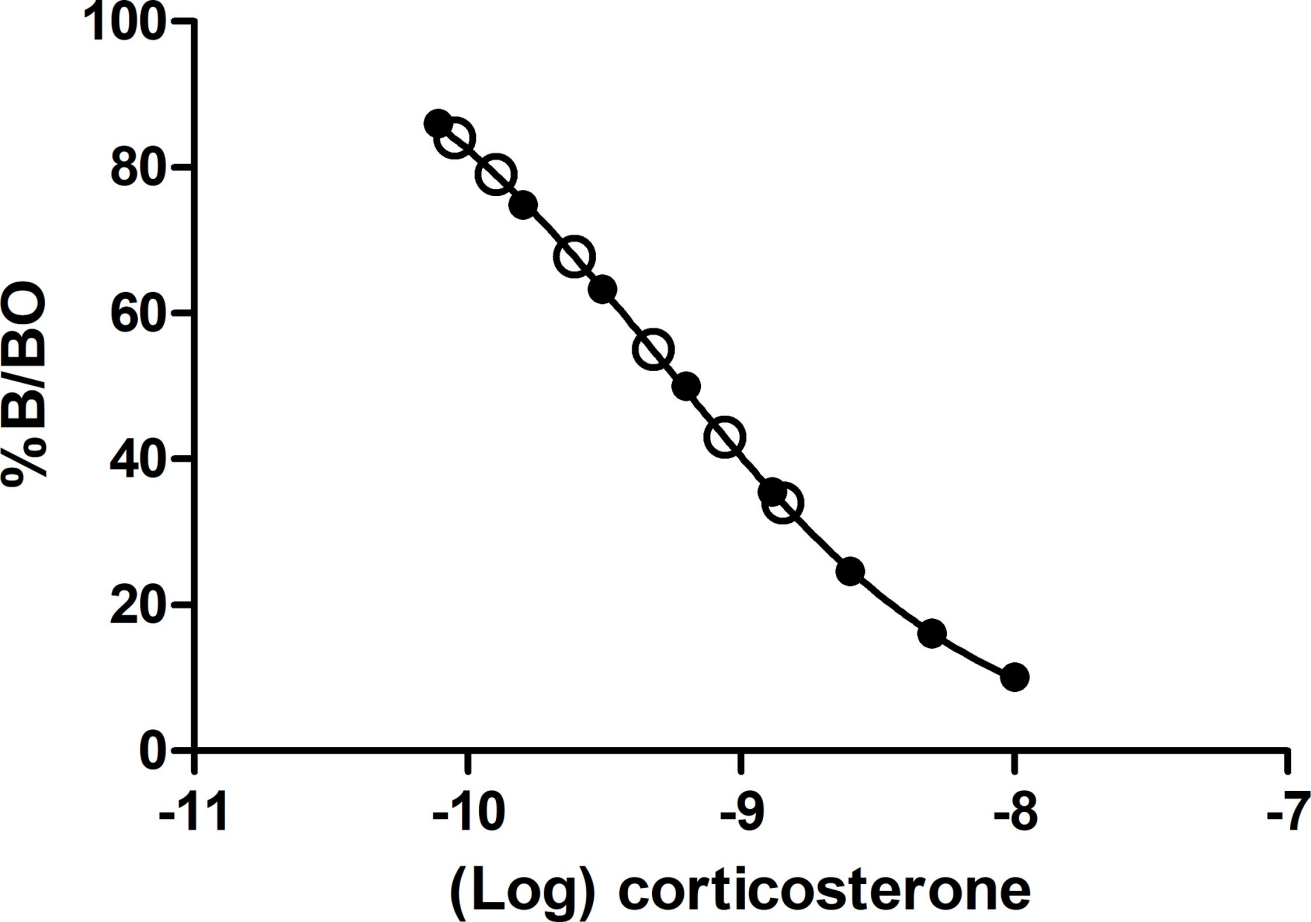
A comparison between the assay kit standard curve (solid circles) and a two-fold serial dilution of metabolized corticosterone pooled together from 10 birds to test for parallelism (Open circles). The x axis shows the Log unlabelled corticosterone concentrations taken from the assay kit and from the pooled sample. The Y axis is the percent of labelled corticosterone %B over the maximum intensity of the assay. There was no difference between the kit standard and pooled sample serial dilution lines (F_1, 10_ = 1.158, P = 0.3).

We collected data in 2015 and 2016 so to ensure that we reduced the possibility of sample deterioration due to excessive storage, we ran the samples in the year they were collected using the following protocols. Total Corticosterone concentrations were measured using an enzyme immune assay (EIA) kit (ARBOR ASSAYS Detect X) which has been validated for use on corticosterone extracted from dry faecal extracts. Before the measurement of samples, we found the assay to be suitable for use on collared flycatcher dropping extracts. To determine this, we pooled together 10 collared flycatcher dropping samples and performed a serial dilution with a series of 8 dilutions although only 6 were detectable by the assay. Serial dilutions of dropping extracts were parallel to the slope of the standard curve provided by the assay kit (Fig 1, N = 10, F_1,10_ = 1.15, P = 0.3). The sensitivity of the assay was determined at 31 pg/ml. We extracted Corticosterone from dropping extracts using an ethanol-based extraction technique as suggested by the kit manufacturer. Dried bird droppings were weighed out to 0.1 g when possible and added to 1 ml of ethanol and shook vigorously for 30 minutes. We centrifuged the samples for 15 minutes at 5,000 rpm then transferred the supernatant to a new tube, before being evaporated to dryness in a SpeedVac. The average Corticosterone recovery was 91.4% ± 1.1% (n = 10). The hormone residue was then quantified using the following method. The assay uses a sheep polyclonal antibody specific for Corticosterone (see S1 Table for the manufacturer steroid cross-reactant test). After evaporation, dried Corticosterone samples were re-dissolved 1:10 with the provided assay buffer. Once reconstituted, 50 μl of each sample was immediately randomly added in duplicates to individual wells on the assay plate. We included all samples relating to the same nest on the same plate (i.e., dropping samples from incubating female and the breeding pair during nestling feeding). All measurements were made using a TECAN infinite F200 PRO plate reader (readings were made at 450 nm). The average inter-plate coefficient of variation was 5.8% ±0.05 (based on two replicates per plate), and the average intra-plate coefficient was 3.25% ± 0.04 (n = 10).

### Statistical analysis

Statistical analyses were performed using the R environment (version 3.3.2(2017)) [34]. First we compared the corticosterone metabolite concentrations to the dropping mass using a linear regression. We also tested the relationship between dropping mass and phase of reproduction using a paired t-test. To test the relationship between the concentration of metabolised corticosterone found in female bird droppings during incubation and the nestling feeding phase, we used a general mixed-effects model with a Gaussian distribution (lmer function in R using the nlme package [35]) and to account for the pseudo-replication caused by the resampling of birds we included female ID as a random effect. We controlled for both female age and hourly recorded air temperature in the model. We then tested the relationship between the level of metabolised corticosterone and air temperature at each stage of reproduction independently using a multiple regression analyses. To meet the assumptions of the constancy of variance and normality, we Log transformed the response variable for both female analyses and square root transformed for the male analysis at the nestling feeding stage. We controlled for the age of the birds in the models. Next, to test the relationship between body condition and corticosterone metabolite concentration at each phase of reproduction in male and female birds we also used a multiple regression analysis. We used a body condition index (BCI) as an estimate of bird body condition and transformed the response variables in each model as mentioned above. We controlled for both bird age and sampling year in the models. Lastly, we tested the relationship between reproductive phase and BCI in female birds as well as the relationship between male and female BCI during nestling feeding using a paired t-test. For all tests, individuals with missing data were removed and the minimal model was identified according to the AIC criteria and stepwise model simplification. Data were presented as means ± 1 SE. Significance was accepted at α = 0.05.

### Body condition index

As an estimate of body condition, we used body mass and tarsus length to calculate a condition index. We opted for the scale mass index (Eq 1) which is a standardization technique [36].

$$\hat{M}i=Mi{\left[\frac{L_{0}}{L_{i}}\right]}^{{ƅ}_{SMA}}$$ Eq 1

The scale mass index standardizes all individuals in the study population to the same growth phase by standardizing the tarsus length and with respect to the scaling exponent (^ƅ^SMA) adjusts the body mass to the new tarsus length. By using the scale mass index, we corrected for any age-dependent effects on body size that might influence the condition score [37]. We used the condition index as a representation of stored energy during the breeding season.

## Results

### Corticosterone metabolites and breeding phase

**Fig 2:**
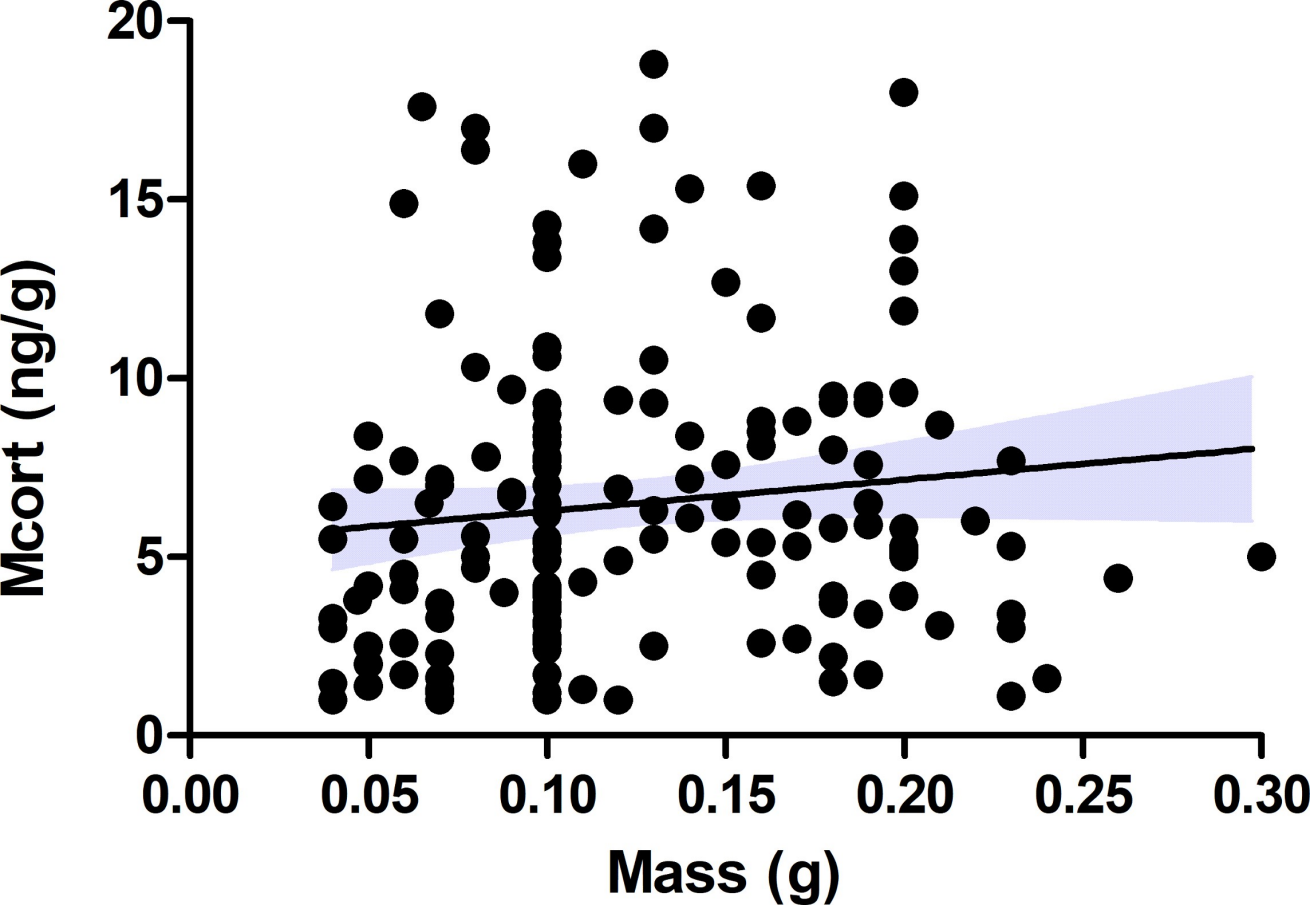
The relationship between dropping mass and corticosterone metabolite (Mcort) concentrations (ng/g). Graph shows fitted line (solid line) and 95% confidence intervals (dashed lines) (N = 167, R^2^ = 0.014, P = 0.12).

**Fig 3:**
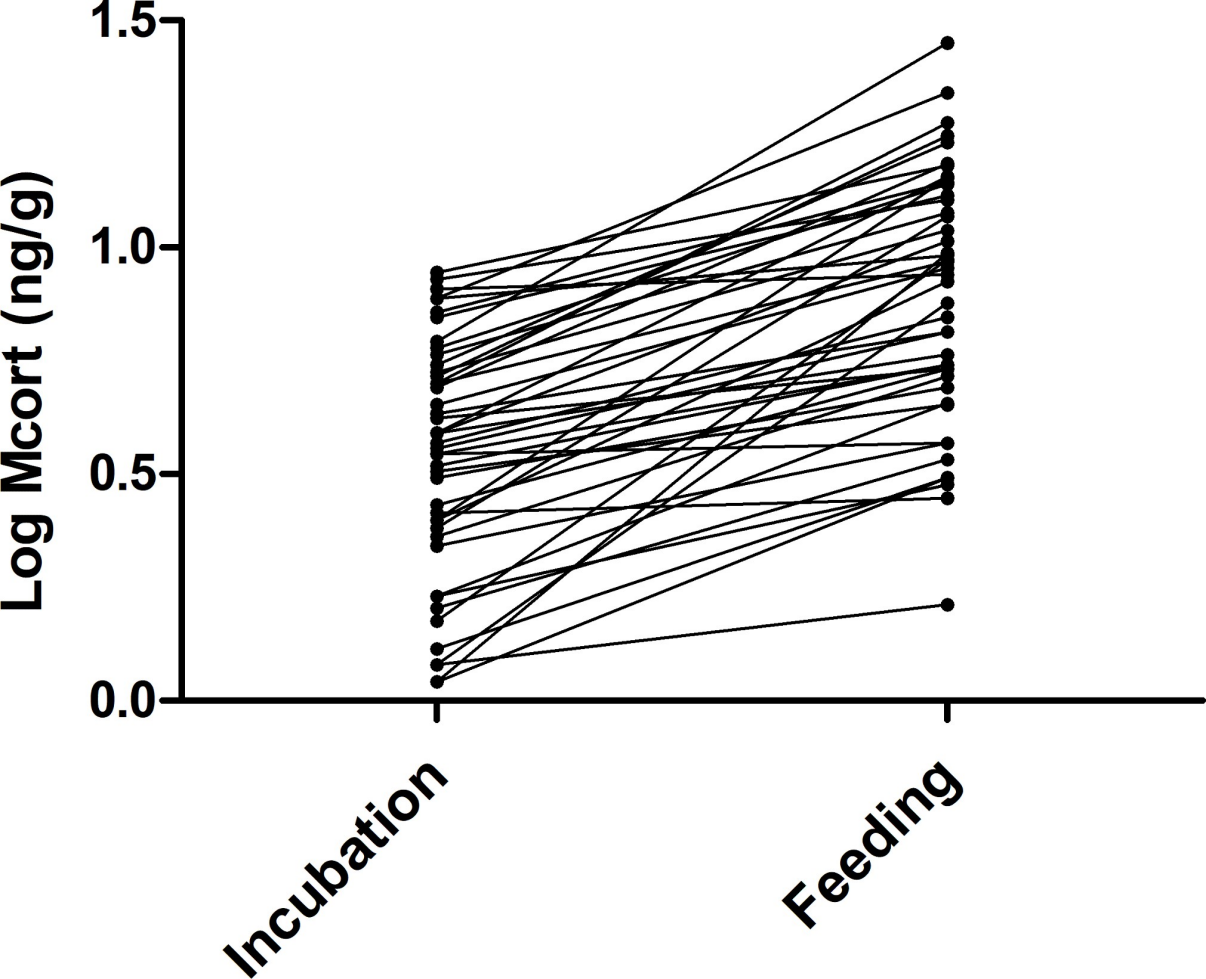
The change in female log corticosterone metabolite (Mcort) concentrations (ng/g) between the incubating and nestling feeding stage (N = 45). The samples were taken five days after the onset of incubation and five days after the nestlings hatched.

We examined corticosterone metabolite concentrations in breeding female collared flycatchers both during incubation and nestling feeding. We did not find that dropping mass related to the phase of reproduction in female birds (t_1,45_ = 0.18, P = 0.85) and there was no significant relationship between dropping mass and concentration of corticosterone metabolites (Fig 2, N = 167, R^2^ = 0.014, P = 0.12). Dropping mass was subsequently excluded from any further analysis. The mean corticosterone metabolite concentration in females during incubation was 4.0±0.26 (ng/g) and 9.4 ±0.84 (ng/g) during nestling feeding (Fig 3, N = 46). In addition to the concentration of metabolised corticosterone detected in females during incubation being significantly lower than during nestling feeding (Fig 3, χ^2^ = 33.2, P = <0.0001) (see S2 Table for model details) the concentration of corticosterone metabolites detected during nestling feeding was highly dependent on the concentration of corticosterone metabolites detected in female bird droppings during incubation (F = 24.9_1, 36_, P = <0.0001). Higher concentrations of corticosterone metabolites during nestling feeding indicates that females expend more energy during this phase than they do during incubation. In addition, the dependence of corticosterone metabolite concentrations during nestling feeding—on the concentrations detected during incubation—is indicative of individual corticosterone phenotypes amongst the birds.

### Corticosterone metabolites and ambient temperature

**Fig 4:**
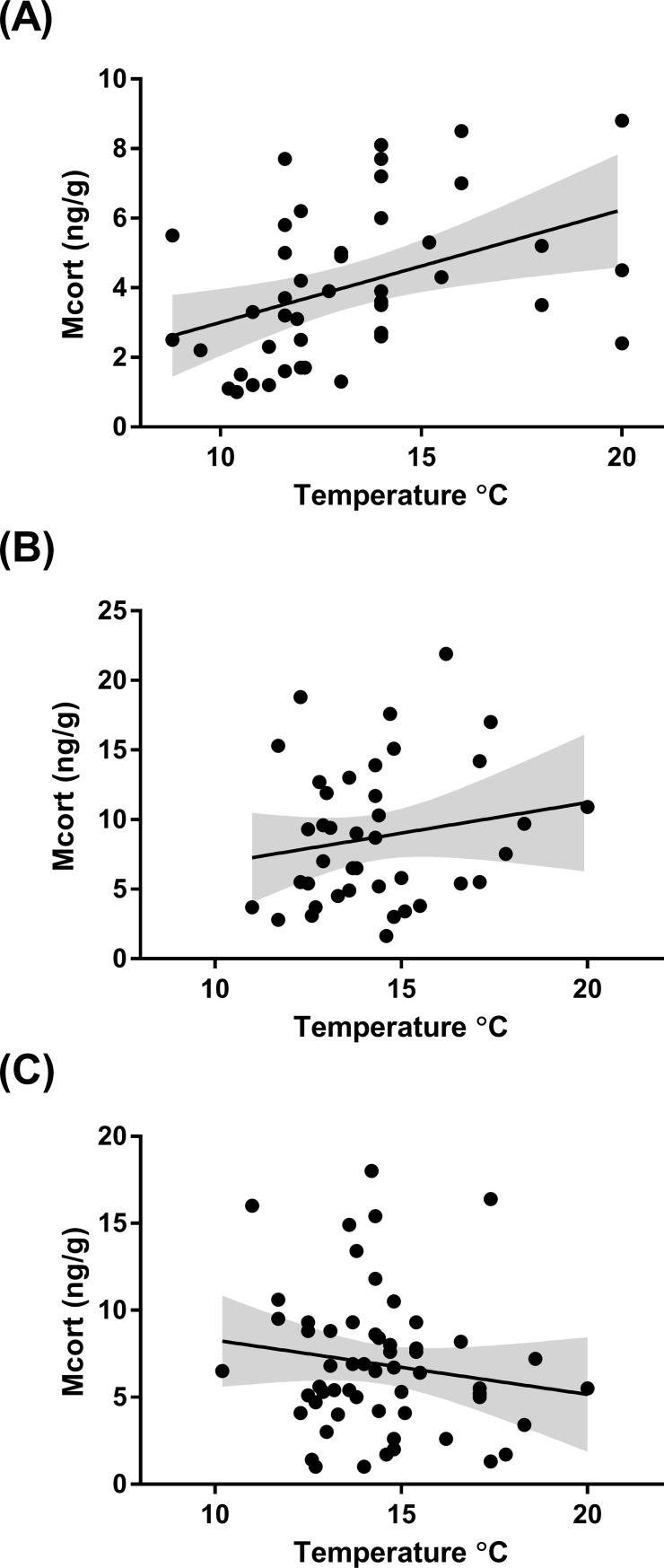
The relationship between metabolised corticosterone (Mcort) concentrations (ng/g) detected in the droppings of (a) female birds during incubation (b) female birds during nestling feeding and (c) male birds during nestling feeding and the mean hourly temperature. Graphs show best fit line and 95% confidence intervals.

**Fig 5:**
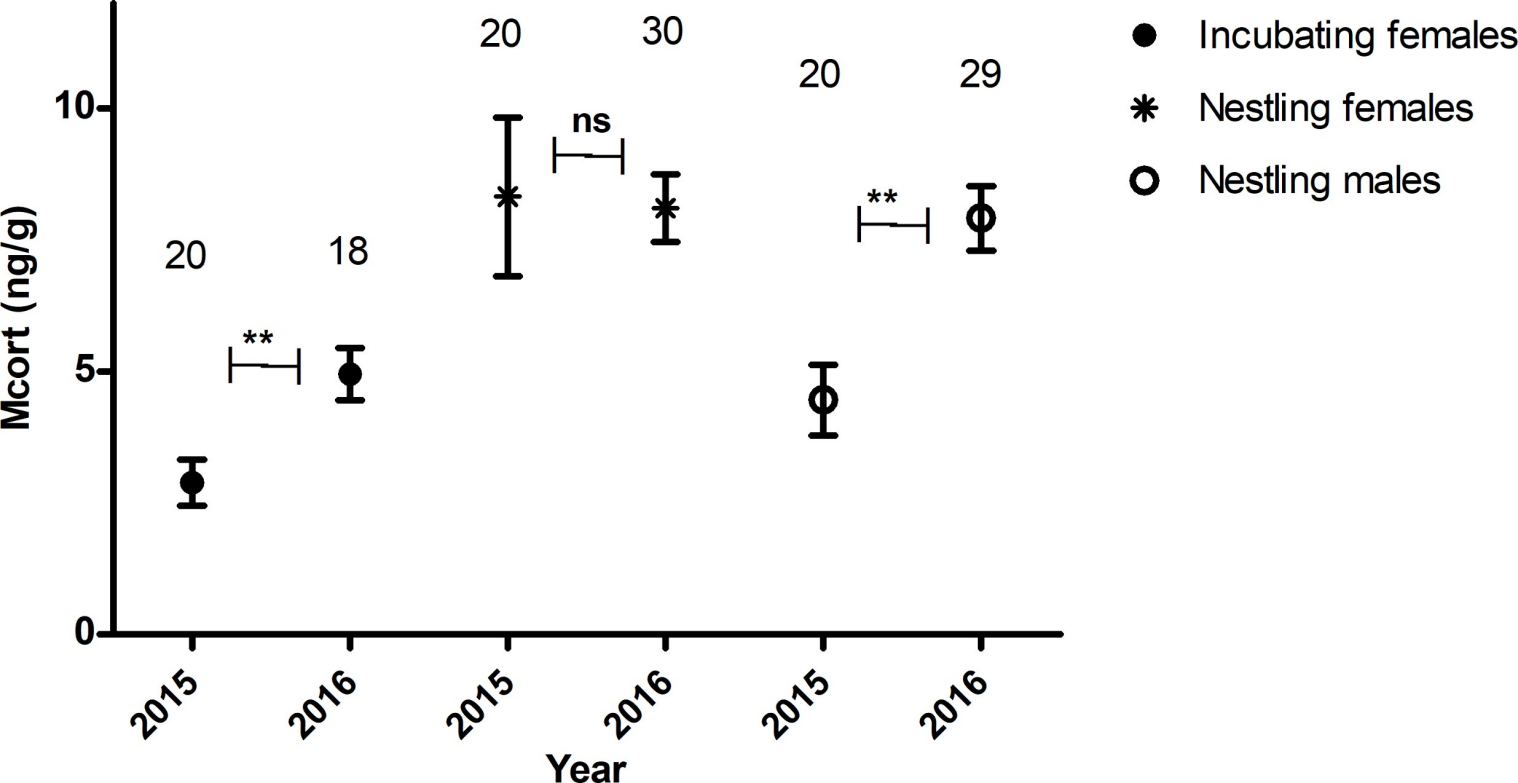
The association between corticosterone metabolite concentrations (ng/g) and sampling year in relation to the phase of reproduction, i.e., females during incubation and males and females during nestling feeding. Points denote the means and bars (SE±). We tested the effect of year on corticosterone metabolites using a multiple regression analysis (See supporting information table D for model details). The significance between years is depicted as not significant (ns), and <0.01 (**). Samples with missing values were removed from the analysis.

Higher ambient temperature was related to higher concentrations of corticosterone metabolites in female birds during incubation (Fig 4A, F_1, 36_ = 5.39 P = 0.025), but no correlation was found during nestling feeding in female (Fig 4B, F = 1.96_1, 36_, P = 0.17) or male birds (Fig 4C, F_1, 56_ = 0.002, P = 0.96) (see S3 Table for model details). Furthermore, the mean temperature over the period of incubation (May-June) was considerably lower in 2015 (8.8 ^o^C) than 2016 (13 ^o^C) which corresponded with a significantly lower mean concentration of corticosterone metabolites in female birds sampled in 2015 (Fig 5, F_1,38_ = 8.07, P = 0.0071). However, although the mean temperature was lower over the nestling feeding period (June-July) in 2015 (12.3 ^o^C) than in 2016 (15.6 ^o^C), only the males sampled in 2015 had on average a lower concentration of corticosterone metabolites than those sampled in 2016 (Fig 5, F_1,56_ = 8.01, P = 0.0064). It is also notable that during the nestling feeding period the precipitation in 2015 (70.8 mm) was approximately twice that in 2016 (35.6 mm). All weather readings were sourced from the Swedish Meteorological and Hydrological Institute (SMHI).

### Corticosterone metabolites and body condition

**Fig 6:**
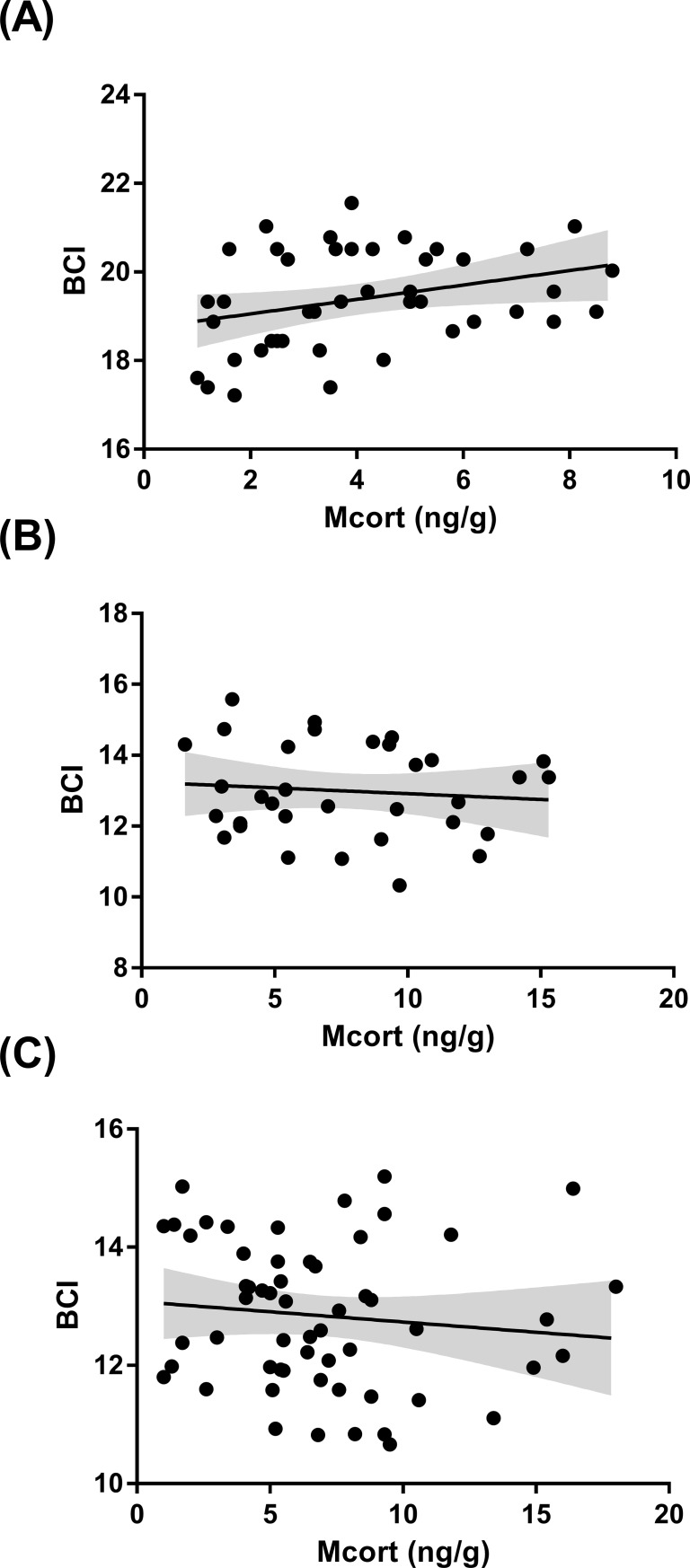
The relationship between body condition using the scale mass index (BCI) in female birds during (a) incubation and (b) nestling feeding and in (c) male birds during nestling feeding and corticosterone metabolite (Mcort) concentrations (ng/g) detected in a single droppings sample. Graphs show best fit line and 95% confidence intervals.

Female BCI dropped significantly from incubation to nestling feeding (t_1,45_ = 31.34, P = <0.0001) indicating that some of the stored energy was being mobilised during nestling feeding. There was a nonsignificant relationship between male and female BCI during nestling feeding (t_1,47_ = 0.25, P = 0.8). Finally, we tested the relationship between the BCI and the concentration of corticosterone metabolites detected in droppings. We found a marginally nonsignificant positive relationship between BCI and corticosterone metabolite concentrations in females during incubation (Fig 6A, F_1, 40_ = 3.75, P = 0.059), but not in females (Fig 6B, F_1,47_ = 0.78, P = 0.38) nor males (Fig 6C, F_1,56_ = 0.53, P = 0.46) during nestling feeding (see S4 Table for model details).

## Discussion

In birds, corticosterone is the primary glucocorticoid responsible for maintaining homeostasis in response to both predictable and unpredictable challenges as they progress through their lifecycle. In this study, we investigated the glucocorticoid response to local conditions and different phases of reproduction by quantifying corticosterone metabolites in the droppings of collared flycatchers. Measuring glucocorticoid metabolites in droppings has the benefit of reducing the effect handling stress has on the level of glucocorticoids measured while also providing a historical measurement from a period of time related to the gut passage [27]. Although, as with all methods of glucocorticoid quantification used in wild population research, measuring glucocorticoids in faeces presents issues including the influence faecal mass has on the concentration of hormone metabolites detected [30]. Here, we detected a change in the level of corticosterone metabolites in collared flycatcher females as a response to a change in energy demand and not dropping mass. Furthermore, we show that measuring corticosterone metabolites is a useful proxy to detect how birds respond to environmental conditions during the breeding season and that this may depend on the current phase of reproduction.

Our finding that corticosterone is upregulated between the phase of incubation and nestling feeding in female birds during the breeding season is consistent with the corticosterone-adaptation hypothesis [21] that predicts the upregulation of corticosterone during the breeding season as part of an adaptive strategy that influences reproductive success. For example, in tree swallows (*Tachycineta bicolor*) fitness was inversely related with corticosterone levels during early incubation, but positively related to corticosterone during nestling feeding. Additionally, house sparrows (*Passer Domesticus*) that had low pre-breeding corticosterone but high breeding levels had better fitness [17], which suggests that the plasticity of the corticosterone response to predictable challenges during the breeding season is crucial for successful reproduction. In this study, all female birds upregulated corticosterone between the incubation and nestling feeding phase (Fig 3) indicating that this is an important strategy facilitating successful reproduction in the collared flycatcher. That said, one must be cautious when drawing such conclusions from corticosterone metabolites because the faecal mass influences the concentrations of metabolites detected in a faecal sample. In an earlier study, it was shown that stonechats (*Saxicola rubicola*) given a diet with increased fibre produced droppings with lower corticosterone metabolites possibly due to an increase in faecal mass [38]. In this study, we do not expect that the difference in corticosterone metabolites detected between the time points to be a consequence of diet. Firstly, because we did not find a relationship between corticosterone metabolites and dropping mass (Fig 2), and collared flycatchers feed entirely on invertebrates during the breeding season. No doubt the availability and type of invertebrates eaten by the birds will vary because of the quality of the breeding territory, foraging ability, environmental conditions and as the breeding season progresses. But, it is unlikely that the significant difference of corticosterone metabolites found between incubation and nestling feeding being a consequence of diet and is more likely a consequence of an upregulation of corticosterone circulating in the blood to meet increased energy demand. A further consideration is the time delay between the stimulation of the HPA-axis and when the subsequent increase in metabolised glucocorticoids is detectable in droppings [27]. We currently do not know the time delay in the collared flycatcher which means that we cannot completely rule out the effect of any stress related incidences on the metabolised corticosterone concentrations quantified. However, the biological significance of the upregulation of corticosterone between incubation and the nestling feeding period again suggests that our result indicates a biological process that facilitates nestling provisioning.

During incubation, the level of corticosterone metabolites in female bird droppings was positively correlated with the current ambient temperature (Fig 4A), and additionally, birds sampled during incubation in 2015, the colder year, produced on average lower corticosterone metabolites than birds measured in 2016 (Fig 5). The cost of incubation is directly linked to current environmental conditions such as ambient temperature that can influence energy expenditure in the form of heat during incubation [14]. And corticosterone regulation, is part of the plastic response that allows birds to adapt to fluctuating conditions such as ambient temperature. For instance, upregulating corticosterone can help to fuel behaviours by initiating the breakdown of stored energy, but if this continues unchecked it can have an adverse effect on body condition and lead to the abandonment of costly behaviours like reproduction [3]. Considering the potential negative consequences of corticosterone, our finding, that corticosterone metabolite concentrations and ambient temperature have a positive relationship is indicative of a mechanism that reduces the breakdown of stored energy when the temperature drops. Thereby, preventing the deterioration of body condition if low temperatures persist, which could ultimately lead to reproduction abandonment [3]. Nest desertion in relatively short lived bird species like the collared flycatcher that have few opportunities to reproduce is likely to be a last resort because each reproductive event is so critical to fitness [39]. Therefore, mechanisms that promote reproduction over reproductive abandonment are likely to be favoured in this species. Furthermore, we found a weak positive relationship between BCI and corticosterone metabolite concentrations during incubation in female birds (Fig 6A), which may similarly indicate a down-regulation of corticosterone to prevent the worsening of body condition. During nestling feeding, however, we did not find a relationship between, ambient temperature or BCI, and the concentration of corticosterone metabolites in female (Figs 4B & 6B) or male birds (Figs 4C & 6C). In a previous study, there was similarly no relationship found between corticosterone metabolites and ambient temperature in either blue tits (*Cyanistes caeruleus*) or pied flycatchers (*Ficedula hypoleuca*) during nestling feeding [13]. The authors suggested that a change in the birds’ behaviour—such as finding shade or more sun exposure—could have counteracted the effect ambient temperature may have had on the corticosterone response otherwise [13], which may also explain our result. In light of this, perhaps the relationship between corticosterone metabolites and ambient temperature in female collared flycatchers during incubation was detectable–as the birds need to incubate the eggs restricts any behaviours that would enable them to adapt to the current ambient temperature. We also suggest that the requirement of elevated levels of glucocorticoids to fuel nestling feeding may have masked any effect that the degree of ambient temperature fluctuations during this study had on the corticosterone response. When comparing female birds sampled in 2015 with those sampled in 2016 during nestling feeding we found that they had on average very similar concentrations of corticosterone metabolites (Fig 5). Whereas males sampled in 2015 during nestling feeding, had on average a lower concentration of corticosterone metabolites than males sampled in 2016 (Fig 5), which was the colder, wetter, season. This means that we cannot rule out that male and female collared flycatchers might have different adaptive strategies to changing environmental conditions during the breeding seasons. The effect of ambient temperature on metabolism can potentially influence the concentration of corticosterone metabolites in faeces because an increase in metabolic rate leads to higher food consumption and distorts what the measurement tells us [30]. Although, as mentioned above, we did not find a relationship between dropping mass and corticosterone metabolite concentration in this study (Fig 2). Similar to the relationship between metabolised corticosterone and ambient temperature we only identified a relationship between BCI and corticosterone metabolites during incubation in female birds (Fig 6A). According to the scale mass index, female body condition drops significantly from incubation to nestling feeding. This reduction in condition is a consequence of the intense workload associated with caring for nestlings which potentially makes condition measurements unreliable at this time.

Our results indicated a dependence between the concentration of corticosterone metabolites detected during incubation and nestling feeding which is suggestive of individual corticosterone phenotypes amongst the females. In future studies, we suggest investigating how the level of corticosterone experienced during early development influences future corticosterone responses to both predictable and unpredictable challenges in adults. Also, we encourage resampling individuals between breeding seasons to determine the repeatability of the corticosterone response as detected as corticosterone metabolites in faeces. In this study, we compared the glucocorticoid response with ambient temperature but other thermal measurements, such as the temperature humidity index (THI) and the wind chill index (WCI), have proved useful when assessing thermal discomfort in animals in relation to the glucocorticoid response [40–43]. Hence, the above mentioned indices provide a potential future direction to determine how collared flycatchers respond to inclement weather.

## Supporting information

S1 Table Assay manufacturer (Arbor assays) steroid cross-reactivity (%). (PDF)

S2 Table The phase of reproduction and corticosterone metabolite quantification. Results of a general mixed effects model assessing the relationship between corticosterone metabolite concentrations (ng/g) and the phase of reproduction in female birds. Corticosterone metabolites were measured in each bird both during incubation and the nestling feeding stage hence we included female identity as a random factor in the model. We controlled for both ambient temperature and bird age in the model.
(PDF)

S3 Table Ambient temperature and corticosterone metabolite quantification. Results of a multiple regression analysis, assessing the relationship between the concentrations of metabolised corticosterone (ng/g) detected in female bird droppings during incubation and male and female birds during nestling feeding and air temperature. Bird age was included in the model to account for any effect it may have had on corticosterone metabolite concentration.
(PDF)

S4 Table Body condition and corticosterone metabolite quantification. Results of a multiple regression analysis, assessing the relationship between the levels of metabolised corticosterone detected in female bird droppings during incubation and male and female birds during nestling feeding and the scale mass body condition index. Also, we tested the effect of the concentrations of corticosterone metabolites (ng/g) detected during incubation on the corticosterone metabolite concentrations detected during nestling feeding in female birds. We included bird age and sampling year in the models to account for any effect they may have had on the concentration of corticosterone metabolites detected.
(PDF)
