# Supplementary material for: Glucocorticoid response to both predictable and unpredictable challenges detected as corticosterone metabolites in collared flycatcher droppings
Source: PLoS One. 2018 Dec 20;13(12):e0209289. doi: 10.1371/journal.pone.0209289 (PMC6301662; doi:10.1371/journal.pone.0209289)
Supplement: S1 Table — (PDF) [file pone.0209289.s001.pdf]

Steroid hormone	Cross-Reactivity (%)
Corticosterone	100%
DesoxyCorticosterone	12.3%
TetrahydroCorticosterone	0.76%
Aldosterone	0.62%
CORTisol	0.38%
Progesterone	0.24%
Dexamethasone	0.12%
Corticosterone-21-Hemisuccinate	< 0.1%
CORTisone	< 0.08%
Estradiol	< 0.08%
