# Supplementary material for: Glucocorticoid response to both predictable and unpredictable challenges detected as corticosterone metabolites in collared flycatcher droppings
Source: PLoS One. 2018 Dec 20;13(12):e0209289. doi: 10.1371/journal.pone.0209289 (PMC6301662; doi:10.1371/journal.pone.0209289)
Supplement: S2 Table — Results of a general mixed effects model assessing the relationship between corticosterone metabolite concentrations (ng/g) and the phase of reproduction in female birds. Corticosterone metabolites were measured in each bird both during incubation and the nestling feeding stage hence we included female identity as a random factor in the model. We controlled for both ambient temperature and bird age in the model. (PDF) [file pone.0209289.s002.pdf]

Random effect V/SD	Fixed effects	Estimate (SD)	X^2	P	DF
7.8/2.7	Stage	5.06(0.72)	33.2	<0.0001	1
	Ambient temperature	0.24(0.16)	2.16	0.14	1
	Age	-0.3(0.4)	0.615	0.43	1
