# Supplementary material for: Glucocorticoid response to both predictable and unpredictable challenges detected as corticosterone metabolites in collared flycatcher droppings
Source: PLoS One. 2018 Dec 20;13(12):e0209289. doi: 10.1371/journal.pone.0209289 (PMC6301662; doi:10.1371/journal.pone.0209289)
Supplement: S3 Table — Results of a multiple regression analysis, assessing the relationship between the concentrations of metabolised corticosterone (ng/g) detected in female bird droppings during incubation and male and female birds during nestling feeding and air temperature. Bird age was included in the model to account for any effect it may have had on corticosterone metabolite concentration. (PDF) [file pone.0209289.s003.pdf]

Sex	Stage	Factor	Estimate (SD)	F	P	DF
Female	Incubation					
		Air temp.	0.07(0.03)	5.39	0.025	1
		Age	-0.07(0.06)	1.29	0.26	1
Female	Feeding					
		Air temp.	-0.06(0.04)	1.96	0.17	1
		Age	-0.11(0.07)	2.16	0.15	1
Male	Feeding					
		Air temp.	-0.002(0.05)	0.0021	0.96	1
		Age	0.0066(0.09)	0.0058	0.93	1
