# Supplementary material for: Glucocorticoid response to both predictable and unpredictable challenges detected as corticosterone metabolites in collared flycatcher droppings
Source: PLoS One. 2018 Dec 20;13(12):e0209289. doi: 10.1371/journal.pone.0209289 (PMC6301662; doi:10.1371/journal.pone.0209289)
Supplement: S4 Table — Results of a multiple regression analysis, assessing the relationship between the levels of metabolised corticosterone detected in female bird droppings during incubation and male and female birds during nestling feeding and the scale mass body condition index. Also, we tested the effect of the concentrations of corticosterone metabolites (ng/g) detected during incubation on the corticosterone metabolite concentrations detected during nestling feeding in female birds. We included bird age and sampling year in the models to account for any effect they may have had on the concentration of corticosterone metabolites detected. (PDF) [file pone.0209289.s004.pdf]

Sex	Stage	Factor	Estimate (SD)	F	P	DF
Female	Incubation					
		BCI	0.154(0.07)	3.75	0.059	1
		Age	-0.07(0.057)	1.83	0.17	1
		Year	0.54(0.16)	8.07	0.0071	1
Female	Feeding					
		BCI	-0.055(0.06)	0.78	0.38	1
		Age	0.0001(0.06)	0	0.9	1
		Inc. cort	0.179(0.03)	4.9	<0.0001	1
		Year	-0.137(0.18)	0.56	0.45	1
Male	Feeding					
		BCI	-0.05(0.07)	0.53	0.46	1
		Age	0.02(0.08)	0.05	0.82	1
		Year	0.56(0.2)	8.01	0.0064	1
